# The Cat: Behaviour and Welfare

**DOI:** 10.1017/awf.2023.44

**Published:** 2023-07-31

**Authors:** Christel Palmyre Henri Moons

**Affiliations:** Animal Behaviour, People & Society Research Group, VIVES University of Applied Sciences, Roeselare, Belgium; Ethology and Animal Welfare Research Group, Department of Veterinary and Biosciences, Faculty of Veterinary Medicine, Ghent University, Merelbeke, Belgium

I felt honoured during the last months of 2022 to be invited by UFAW to review the book, *The Cat: Behaviour and Welfare* from the well-respected scientists and authors Bjarne Braastad, Anne McBride and Ruth Newberry. The time (too long, unfortunately) it took to write the review was partly due to Brexit and the fact that books just do not travel as easily anymore from the UK to Europe. Another reason was that, despite the kind efforts of UFAW and CABI to supply me with a digital copy while awaiting arrival of the shipment, I paused my reading to receive the paper copy. Holding the book physically, I could leaf back and forth without swiping, clicking, Ctrl-F-ing or any other digital action, adding to my reading and reviewing enjoyment!

“Another book about cats, really?”, you may think. And you would be right, were it not for the fact that the book by Braastad and colleagues does more than simply offer up a comprehensive overview of what is already written in other books. True, this book does supply classic chapters such as the ones on the origin of cats, cat communication and problem behaviours in cats. However, it gives an update on those topics where possible, and also approaches cats from more contemporary angles, like cat personality (an aspect that is quickly gaining momentum in animal welfare science) and particularly the individual variation that must be considered alongside breed variation, the relationship between people and cats (including COVID and the lessons learned there), and how cats contribute to human health. I found that many chapters had nice surprises of content I was not expecting. The book in its entirety is a comprehensive combination of scientific knowledge, anecdotal information, and practical advice for cat owners, although the authors state that the book is also useful for others interested in the lives of cats and in caring for them (and I would agree).

Personally, as a scientist interested in cat behaviour and welfare and as a cat aficionado, I can only applaud more books that divulge knowledge about keeping and caring for cats, if they are as enjoyable to read and scientifically sound as this one. The content is, as the authors promised in their preface, apt for those who do not yet know much about cats, but also allows for deepening of knowledge for those who do. The text reads fluently, and the content is captivating. As a scientist, I did at first get a bit annoyed with the many personal anecdotes that are dispersed throughout the book, until I remembered that anecdotes are a cornerstone of how scientific research develops. As soon as that sunk in, I dove into every anecdote, thinking more about it and what it could mean. Also, the reader may wonder why there are several text-boxes devoted to national legislation and good practice guidelines (mostly from Norway and the occasional one from the UK). This no doubt reflects the nationality of certain authors and their familiarity with the information available in their country, but it did feel somewhat selective, since progressive legislation and good practices exist in other countries, too. Finally, I appreciated the supplemental audio and video content that is offered throughout the book via QR codes. Unfortunately, scanning those in my edition of the paper version, resulted in “404 – page not found” error messages, a problem I was able to circumvent by accessing the web page directly through a Google search: https://www.cabi.org/products-and-services/about-cabi-books/open-resources/the-cat-behaviour-and-welfare/. The digital edition of the book did not have these QR code issues.

The book contains 14 chapters and, appropriately, the first starts with the origin of the cat, dispersal of the cat, and a brief discussion on the genetic relatedness of cat breeds. The next chapter discusses cat reproduction and the development of kittens, both physically and behaviourally. It provides ample advice for breeders and new owners to ensure a kitten has the best chance at a good physical and mental (cognitive and emotional) development. It also highlights the importance of ID marking and vaccination. I particularly appreciate the part that discusses the fact that a new cat owner should have sufficient knowledge (of the correct kind) to care for a cat and that it is a breeder’s responsibility to ensure that this is the case.

Chapter 3 discusses the factors contributing to the individual characteristics of cats, such as genetic make-up, age, gender, and breed. Some interesting Norwegian master theses studies are discussed containing information that would otherwise be unavailable to a non-Norwegian-speaking audience. What I do tend to miss at times is a more careful interpretation of certain results or additional information regarding the studies, allowing the non-lay audience to better evaluate the impact of the results. But this will be of no importance to the main audience (cat owners) and the information provided is sufficiently clear and always useful.

Chapters 4, 5 and 6 discuss some aspects of cat behaviour, respectively, communication, social behaviour and one of the hallmarks of being a cat, i.e. predation. These chapters also provide a large knowledge base and a comprehensive overview. In the communication chapter, the presentation of acoustic communication caught my eye. Cat communication in general is not always easy to observe and interpret correctly. To interpret miaowing, the authors refer to the system described by Mildred Moelk who interprets calls according to where the emphasis on ‘miaow’ is placed. Audio fragments are supplemented to distinguish the different calls. It is a useful framework, although I can imagine that some readers will confuse ‘Miaaao’ with ‘Miaooww’ still with respect to their meaning, assuming that operant conditioning will shape the way an individual cat vocalises to its owner. In the chapter on social behaviour of cats, I appreciated the inclusion of advice for multi-cat households, as this often artificial situation (getting another cat is more often than not the choice of the owner, not of the resident cat) can lead to severe welfare problems for both resident and newcomer cats.

My favourite chapter was perhaps chapter 7 on the cat’s ability to navigate. Rarely is this topic discussed in much depth and getting an insight into the existing scientific research was a real treat for me. I assume the same will be true for many cat owners who often wonder about their freely roaming cat. It is seamlessly followed by advice on what to do when you lose your cat (spoiler alert: the preventive measure of ID tagging your cat is emphasised too), whether you should take your cat on holiday with you, and the best practices should you decide to do so. However, since we return to the latter topic in chapter 9, perhaps it would have been better to have moved and integrated the information into there, to avoid unnecessary confusion for the cat owner wishing to make an informed decision about holidays and their cats.

Chapter 8, explaining motivation, behavioural needs and emotions is an important precursor to chapter 9, about cat welfare. It is good to see that we have moved away from animal welfare as only involving the avoidance of negative emotional states in animals and have expanded the concept to also procure positive emotional states in animals. The fact that animal welfare is about the state of the animal as experienced by the animal, and not about what we as humans think is good for animals, cannot be emphasised enough. On a less theoretical note, this chapter also addresses the issue of homeless cats, environmental choices for cats, and another touch upon what to do when you go on holiday (with an extension on requirements for cat boarding and cat shelters).

Chapter 10 addresses learning and training in cats, which is important in its own right, but also has relevance as a background for chapter 11 on behavioural problems. For some cat owners, learning theory can create confusion (especially the difference between positive/negative reinforcement/punishment), but this chapter provides an accessible overview of the key points. Chapter 11 helps the owner distinguish who the behaviour is a problem for (which can sometimes indicate the urgency with which it should be addressed or, instead, the owner should be educated about natural cat behaviour). Both preventative and curative actions are discussed, with the most important advice given being that owners should not hesitate to contact a veterinarian and/or a qualified behaviourist when they wish to address unwanted behaviour in their cat. Unfortunately, professionalisation of the animal behaviour counselling field is still problematic in many countries and the quality of advice cannot always be relied upon. Therefore, it is still useful that the reader is provided with an overview of the most common behavioural problems, the emotions driving them, and a starting point of how to go about addressing them. Such information will also be helpful when speaking to a professional.

Chapters 12 and 13, titled ‘People and Cats’ and ‘The Cat’s Contribution to Human Health’, respectively, deal with the human-cat bond and the benefits thereof. The first chapter focuses on the complexity of the relationship, how cat-keeping is changing over time and place, and which factors play a role (not least the aspect of financial means). Attention is also being paid to the lessons learned from the COVID pandemic. Given the concepts of One welfare and One Health, both chapters represent a welcome addition to a contemporary book about cat-keeping and caring.

Chapter 14 is a concluding chapter that provides an informative summary about raising and keeping cats that function well in a society of people. And here I would like to conclude as well, by applauding the authors for having written a book that is not only meant to inform cat owners about cat behaviour and welfare but also seeks to help them take good care of their cat. As someone who sees “where things go wrong with cats” or at least “where we could do better” on almost a daily basis, I appreciate that the text also continuously emphasises the responsibility owners have towards the cat they have acquired. In my opinion, this book is an important contribution to a much-needed shift in the attitude of people towards cats specifically and animals in general, from a culture of ownership and anthropocentrism to a culture of care. I hope it can get translated into a few different languages in future…

